# Three-Dimensional Speckle Light Self-Healing-Based Imaging System

**DOI:** 10.1038/s41598-017-18952-0

**Published:** 2018-01-12

**Authors:** Danilo G. Pires, Artur F. Sonsin, Alcenísio J. Jesus-Silva, Eduardo J. S. Fonseca

**Affiliations:** Instituto de Física, Universidade Federal de Alagoas, Maceio, Alagoas 57061–970 Brazil

## Abstract

Recently new methodologies for imaging have been achieved making use of multiple light scattering. Here we present the self-healing effect using a speckled light field. We present an experiment that constitutes a useful application for a three-dimensional light sheet-based imaging system through an inhomogeneous medium. Each layer can be imaged independently of the others. The axial resolution basically depends on the coherence length, which can be sub-wavelength and controllable. This allows for a simple and direct technique for imaging through scattering layers with axial resolution improvement. Our results may find applications not only in bio-microscopy systems but also in data transmission.

## Introduction

The coherent laser is very sensitive to the scattering, however a well-known class of no-diffracting beams, known as Bessel beams, has shown robustness to scattering due to the self-reconstruction phenomenon. This particular beam has been largely used in several applications, including contrast enhanced imaging and increased penetration depth in dense media^1–3^, optical manipulation^4^, optical coherence tomography^5,6^ and quantum correlation^7^. In bio-photonics, the self-reconstruction phenomenon with coherent Bessel beams has opened new research applied to the modern life science which often requires three-dimensional (3D) imaging^8^. It’s worth emphasizing that other beams present the non-diffractive property like Mathieu beams^9^ or even speckles^10^, and the self-healing is a property not limited to non-diffracting beams^11^.

On the other hand, the speckle field, which consists of random dark and bright spots because of a multiple scattering processes^12^, is present in a variety of applications, especially in imaging system. In fact, nowadays there is already a well-established technique called Structured Illumination Microscopy (SIM)^13,14^ that use speckles as a light source. Additionally, some works have explored speckles for microscopy in biological samples^15–18^, in photo-acoustic imaging^19^, and passive near-field imaging^20^. One important feature in these experiments is the improved resolution and elimination of typical imaging artifacts. The main point for most of the above mentioned applications is how to extract the information from the speckles. All procedures try to find the best optimized algorithm to descramble the information contained within the speckles.

Here we present a 3D microscopy system where it is possible to obtain independent images of superimposed planes in a direct way, without the necessity of complex computational algorithms to descramble the images and stringent system calibrations. The idea is based on the self-reconfiguration effect^21^, where the speckles after being partially blocked can be reconfigured after the reconfiguration length^21,22^. Using this principle, it is possible to explore most of the applications that has been done with Bessel beams but with speckles features. Particularly, it is possible to overcome Bessel beams in imaging applications.

## Methods

**Figure 1:**
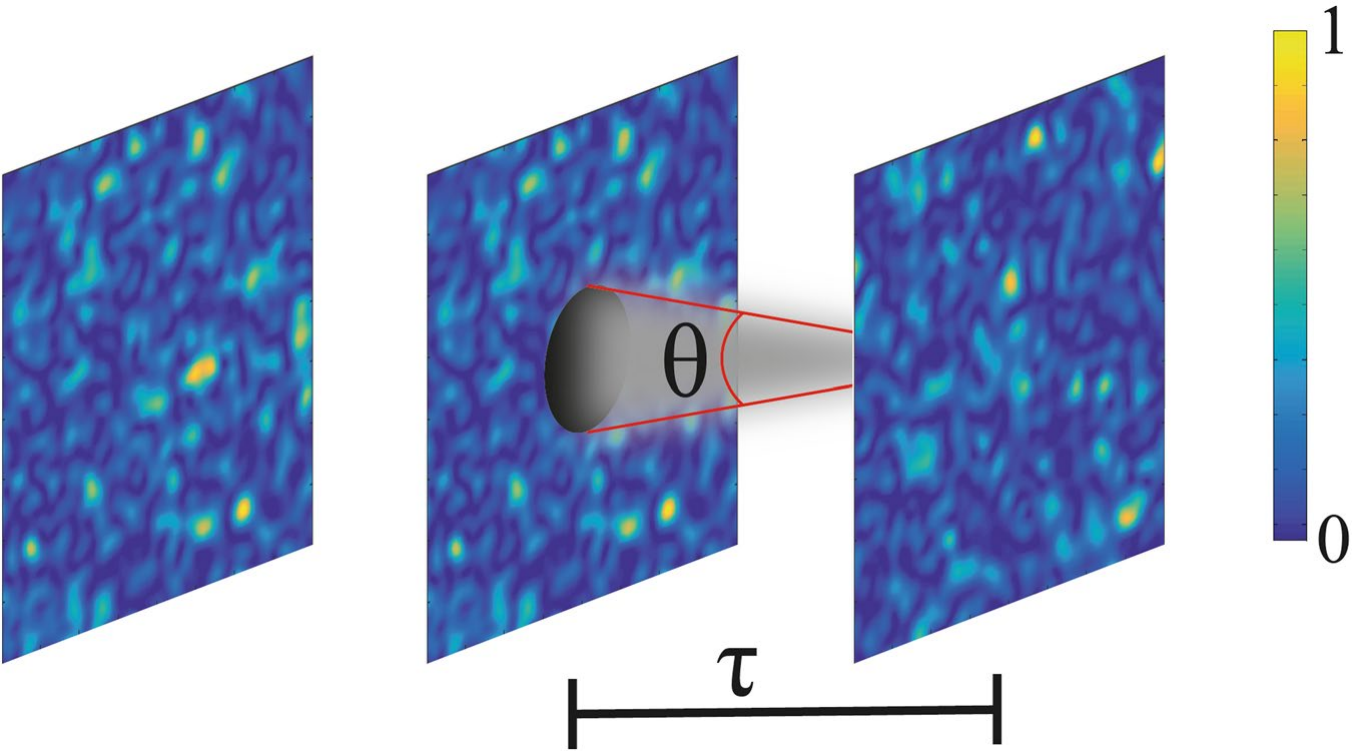
A speckle field crossing an obstruction and becoming homogeneous after the reconfiguration length given by Eq. 1.

Recently, a study showed that a speckle pattern can be reconstructed after being obstructed by an obstacle^21^. An object is placed on the path of the speckles and after a specific distance, called reconfiguration length, the signature of the object is completely washed out. The speckle pattern becomes homogeneous. This is the so-called self-reconfiguration effect^21^. Additionally, a theoretical model was presented with an explicit equation to the reconfiguration length τ^22^,

1 $$\tau =1.93\frac{\delta D}{\lambda }$$

where δ is the transverse coherence length, λ is the wavelength of the light source and *D* is the disc diameter. One can explain the self-reconfiguration effect through the Huygens’ Principle. Suppose an opaque obstacle is placed in the beam path as shown in Fig. 1. The spots surrounding the obstacle will act as a secondary source that radiates after the obstacle and, after the reconfiguration length, a new speckle pattern appears without the signature of the obstacle. The angular spread of a spot can be written in terms of the transverse coherence length *δ* and the wavelength λ as $\theta \,\propto \,\lambda /\delta$. Assuming small divergence, the portion of the spots that is not blocked by the obstruction reconstructs the speckle pattern beyond the shadow of the disk at a distance $z=D/\theta \propto \delta D/\lambda$, where *D* is the disc diameter, resembling Eq. 1^22^. An interesting point though is that, we can align as many different opaque objects as we want along the beam axis and separated by a distance $d\ge \tau$, and after τ from the last object, a homogeneous speckle pattern will be observed.

**Figure 2:**
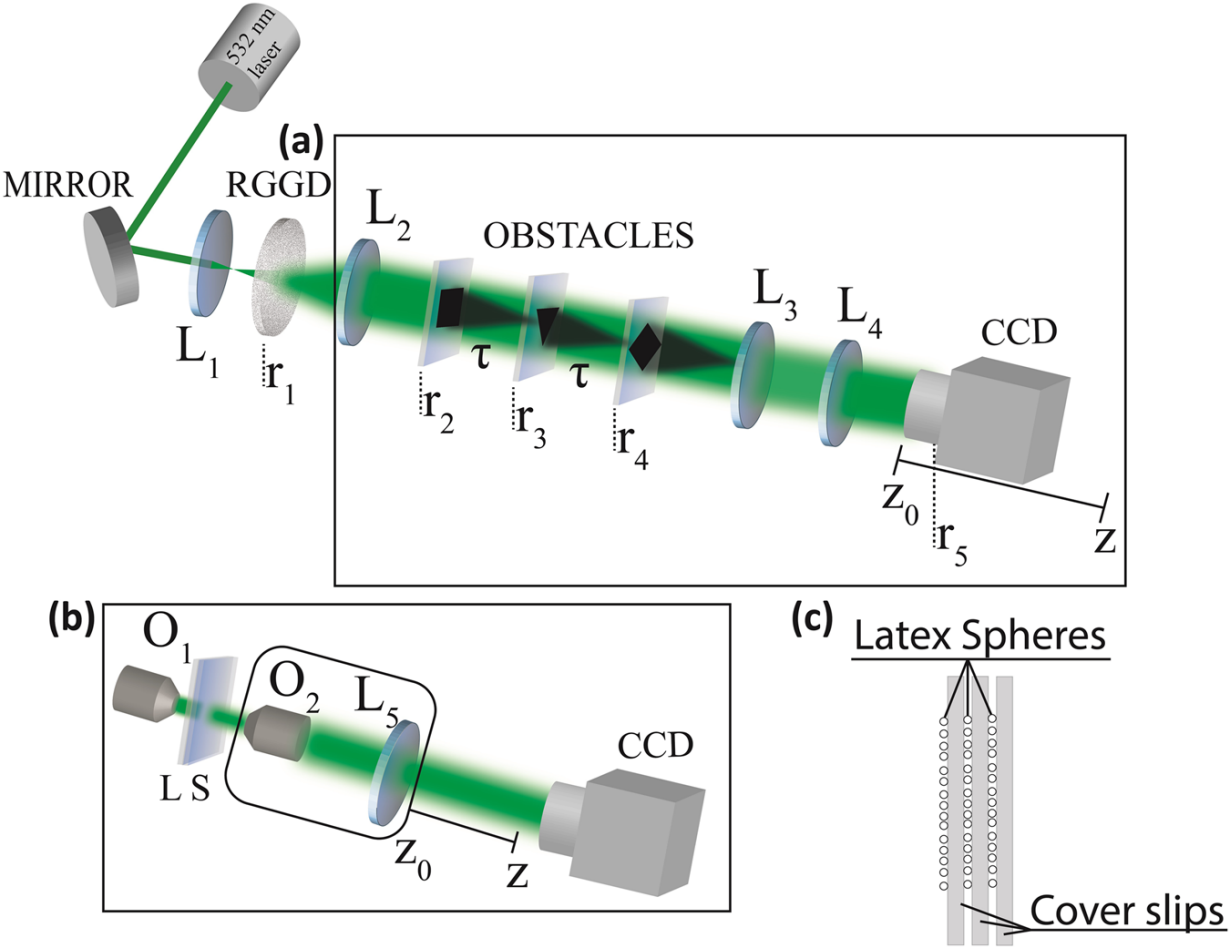
Experimental setup, where *L*_*i*_, $i=1,2,3,4$ and 5 are lenses, *O*_1_ and *O*_2_ are objective lenses, RGGD is the rotating ground glass disk and CCD stands for charge coupled device camera. Two configurations were used in this paper for different samples: (**a**) three opaque objects and (**b**) latex spheres sample. (**c**) Illustrative picture showing three glass cover slips with the latex spheres samples.

Now, instead of worrying about to erase the signature of the opaque objects, we will focus on seeing their images. In order to implement that, imaging and detection systems must be aligned after the last object. The idea is described in Fig. 2(a), where three opaque objects are aligned distancing from each other of τ. The plane of the first object, a dark square, is projected by the imaging system into the CCD camera at position $z=0$, consequently a dark square should be observed. Interesting enough, if the CCD camera is moved of $z=\tau$, the second object, a dark triangle is clearly observed without any signature of the first one, as we will show later on. In addition, we will see clearly a lozenge if the detector is moved, now to the position $z=2\tau$, without any signature of square or triangle. In principle, we could have a microscopy system that could see planes behind of opaque objects, where the axial resolution is limited by the reconfiguration length, i.e., by Eq. 1.

## Results

### Experiment

**Figure 3:**
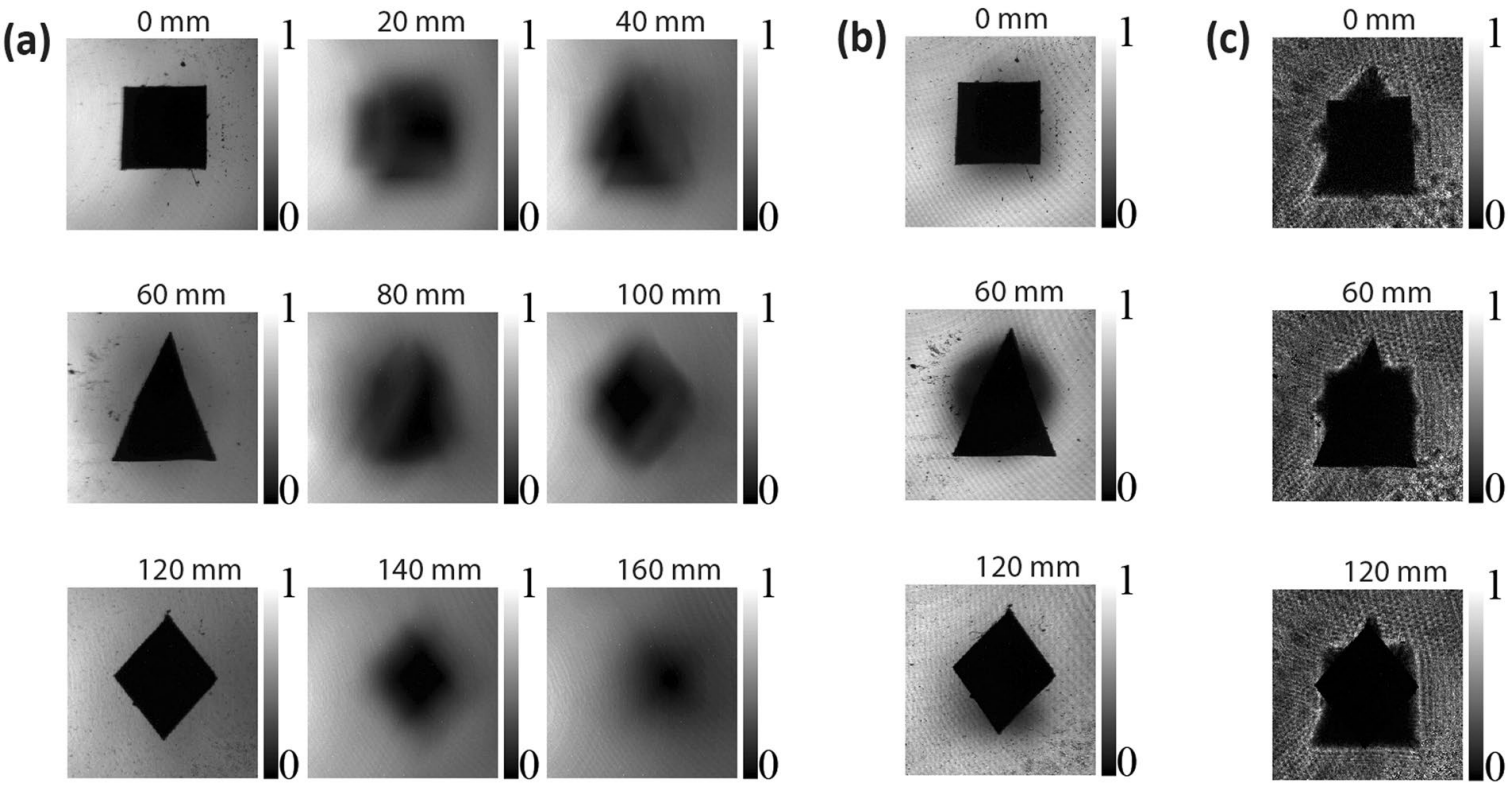
Mean intensities direct detect by the CCD camera at different longitudinal positions, where the used coherence lengths were (**a**)$\,\delta =7.2\,\mu m$, (**b**) $\delta =23.8\,\mu m$ and (**c**) completely coherent beam. To obtain the images in the experimental results we have used two type of averaging simultaneously: temporal averaging due to the integration time of the CCD camera while the RGGD was kept rotating and the averaging over different captured images. The exposition time of the CCD camera (**a**), (**b**) was $300\,ms$ and an average over 50 images was performed.

As illustrated in Fig. 2(a), to produce the speckled light source a Nd:YAG laser operating at 532 *nm* is transmitted through a RGGD. In this figure, a lens *L*_1_, with focal length of ${f}_{1}=11\,mm$, was used to control the size of the incident laser beam on the RGGD. By moving *L*_1_ longitudinally, the spot size of the laser beam on the disk changes accordingly, allowing to control the mean speckle grain diameter, which is roughly the coherence length^23^. Three objects were aligned in the beam path: a dark square, triangle and lozenge, all with the same area of $4\,m{m}^{2}.$ A pair of confocal lenses *L*_3_ and *L*_4_, of focal lengths ${f}_{3}={f}_{4}=f=200\,mm$, was used to image the objects at a CCD camera with unit magnification. A lens *L*_2_ was placed at its focal distance ${f}_{2}=140\,mm$ from the RGGD to collimate the speckles. Figure 3 shows the experimental results for the normalized intensity patterns recorded by the CCD camera for the configuration of Fig. 2(a). In this case, we have moved the CCD camera along the longitudinal position written on the top of the images. To obtain the images showed in Fig. 3(a,b), we have used temporal averaging, due to the integration time of the CCD camera while the RGGD was kept rotating. These images were recorded with an exposure time of *3*00 *ms* to each captured image and an average over 50 images was performed. Figure 3(c) shows images acquired using a coherent beam. In this case, the RGGD was removed of the beam path. Figure 3(a) shows perfect images of a square, a triangle and a lozenge at *0*, *60* and *120 mm*, respectively. In the range *0–60 mm* some blurry images are observed as a superposition of a square and a triangle, evolving to a clear triangle image at *60 mm*. The same effect can be observed in the range *60–100 mm*, but now, the images develop to the lozenge. Here, the obstacles were placed at *60 mm* from each other matching the reconfiguration length, and the images were acquired by longitudinally moving the CCD camera. The speckle coherence length used to obtain the images presented in Fig. 3(a) was $7.2\,\mu m$. Figure 3(b) shows superimposed images of three objects. However, at the positions *0*, *60*, and *120 mm* the images of the square, the triangle and the lozenge are more evident, respectively. A small superposition happened because the coherence length used for this case was of $23.8\mu m,$ which correspond to a reconfiguration length of *160 mm*, bigger than the object separation of *60 mm*. The results presented in Fig. 3(b) highlight the fact that the distance between the objects cannot be smaller than the reconfiguration length in order to acquire a sharp image. In Fig. 3(c), we have used a coherent beam where all images are superimposed over each other. The results presented in Fig. 3 show that the reconfiguration length indeed controls the axial resolution of the imaging system.

### Analytical approach

A theoretical model was coined for the configuration presented in Fig. 2(a), but contains the basic operating principle. The field impinging on the RGGD is a field whose transversal profile is Gaussian,

2 $${E}_{1}({\overrightarrow{r}}_{1})=\exp (-{r}_{1}^{2}/{w}_{0}^{2}\,)\exp (i\psi ),$$

where ${w}_{0}$ is the beam waist and $\psi$ its phase. ${\overrightarrow{r}}_{i}$ $(i=1,2,3,4,5)$ represents the transversal coordinates for different propagation distances following the sketch of Fig. 2(a). The description of the field propagation from the RGGD to the first obstacle (square) is given by a Fourier transform,

3 $${E}_{2}({\overrightarrow{r}}_{2})=\int {E}_{1}({\overrightarrow{r}}_{1})G({\overrightarrow{r}}_{1})\exp (\frac{i2\pi }{\lambda {f}_{2}}{\overrightarrow{r}}_{1}\cdot {\overrightarrow{r}}_{2}){d}^{2}{r}_{1},$$

where $G({\overrightarrow{r}}_{1})$ describes de effect of the RGGD and λ is the wavelength. Successively applying the Colins’ integral formula^24^, we can describe the field propagation between the obstacles and lenses to the CCD camera plane. At the end we have,

4 $$\begin{array}{c}{E}_{5}({\overrightarrow{r}}_{5})=\int L({\overrightarrow{r}}_{4})\exp [\frac{i\pi }{\lambda (2d-z)}{({\overrightarrow{r}}_{4}+{\overrightarrow{r}}_{5})}^{2}]\int T({\overrightarrow{r}}_{3})\exp [-\frac{i\pi }{\lambda d}{({\overrightarrow{r}}_{3}-{\overrightarrow{r}}_{4})}^{2}]\int S({\overrightarrow{r}}_{2})\exp [-\frac{i\pi }{\lambda d}{({\overrightarrow{r}}_{2}-{\overrightarrow{r}}_{3})}^{2}]\\ \quad \quad \quad \quad \times \int {E}_{1}({\overrightarrow{r}}_{1})G({\overrightarrow{r}}_{1})\exp (\frac{i2\pi }{\lambda {f}_{2}}{\overrightarrow{r}}_{1}\cdot {\overrightarrow{r}}_{2}){d}^{2}{r}_{1}{d}^{2}{r}_{2}{d}^{2}{r}_{3}{d}^{2}{r}_{4},\end{array}$$

where $L({\overrightarrow{r}}_{4})$, $T({\overrightarrow{r}}_{3})$ and $S({\overrightarrow{r}}_{2})$ represent the lozenge, triangular and squared obstacles, respectively, and z.is the displacement of the CCD camera from its initial position at the focus of *L*_4_. The mean intensity $I({\overrightarrow{r}}_{5})=\langle {E}_{5}({\overrightarrow{r}}_{5}){E}_{5}^{\ast }({\overrightarrow{r}}_{5})\rangle$ detected by the CCD camera shows exactly the images in Fig. 3 when the corresponding limits are taken into account. For the case $z=0$, the mean intensity leads to $I({\overrightarrow{r}}_{5})\propto S(-{\overrightarrow{r}}_{5}).$ For the case $z=d$ we observe the triangle obstacle, or $I({\overrightarrow{r}}_{5})\propto T(-{\overrightarrow{r}}_{5})$. The last case is for $z=2d$, which leads to $I({\overrightarrow{r}}_{5})\propto L(-{\overrightarrow{r}}_{5}).$ The minus sign in the transversal coordinates of the intensity patterns means that the images are inverted. The full analytical treatment is available in the Supplemental Material.

### Latex Spheres

In order to give an overview of how useful can be the concept behind the results shown in Fig. 3, we present a new experiment with the configuration shown in Fig. 2(b). Three glass cover slips with approximately $0.08-0.13\,mm$ of thickness and containing latex spheres were aligned between the two objectives lens,*O*_1_ and *O*_2_, of focal lengths ${f}_{O1}=0.13\,mm$ and ${f}_{O2}=4.5\,mm$, respectively. The objective lens *O*_2_ and the lens *L*_5_ which has focal length ${f}_{5}=100\,mm,$ were mounted in a translating stage and can be moved along the longitudinal position z. By moving this stage, it is possible to observe independent microscopic images of longitudinally superimposed layers. The average latex spheres diameter was of 3*μm*. We aligned the samples as illustrated in Fig. 2(c).

**Figure 4:**
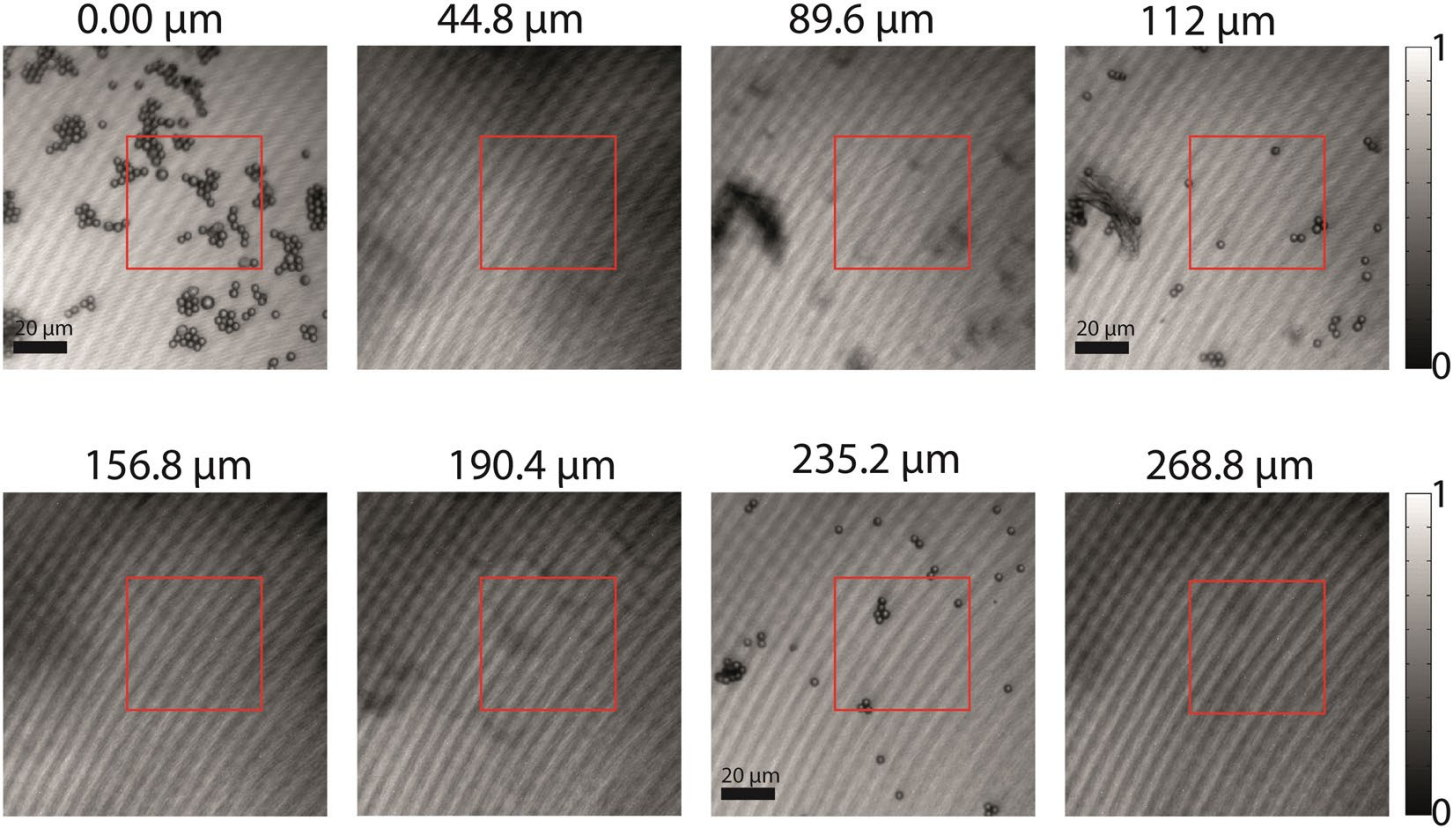
Experimental results for imaging of latex spheres. Images obtained using mean intensity of laser speckles illuminating the sample with three-layers of latex spheres. The values on the top of the figures corresponds to the longitudinal displacement of the set $O2-{L}_{4}$. We have used an exposition time of 300 *ms* and averaged over 50 captured images.

Figure 4 shows some of the images referring to the different latex spheres planes with the *z* position indicated on top of the images. In the supplementary material a movie of all layers can be seen. It is clear that the pictures at the axial coordinates 0, 112 and 235.2 *μm* present sharp images of the spheres and between these positions only some blurry images of them or just the interface of the glass sheets are seen. The coherence length of the speckle pattern at the sample was of the order of 1 *μm* Notice that the distance between the planes of the sharp spheres images is much bigger than the reconstruction length. In fact, we have shown that it is possible to image different longitudinal planes without superposition of the images. The latex spheres in one layer or even the roughness of the substrate that contain the spheres do not distort the images of the individual planes of spheres.

**Figure 5:**
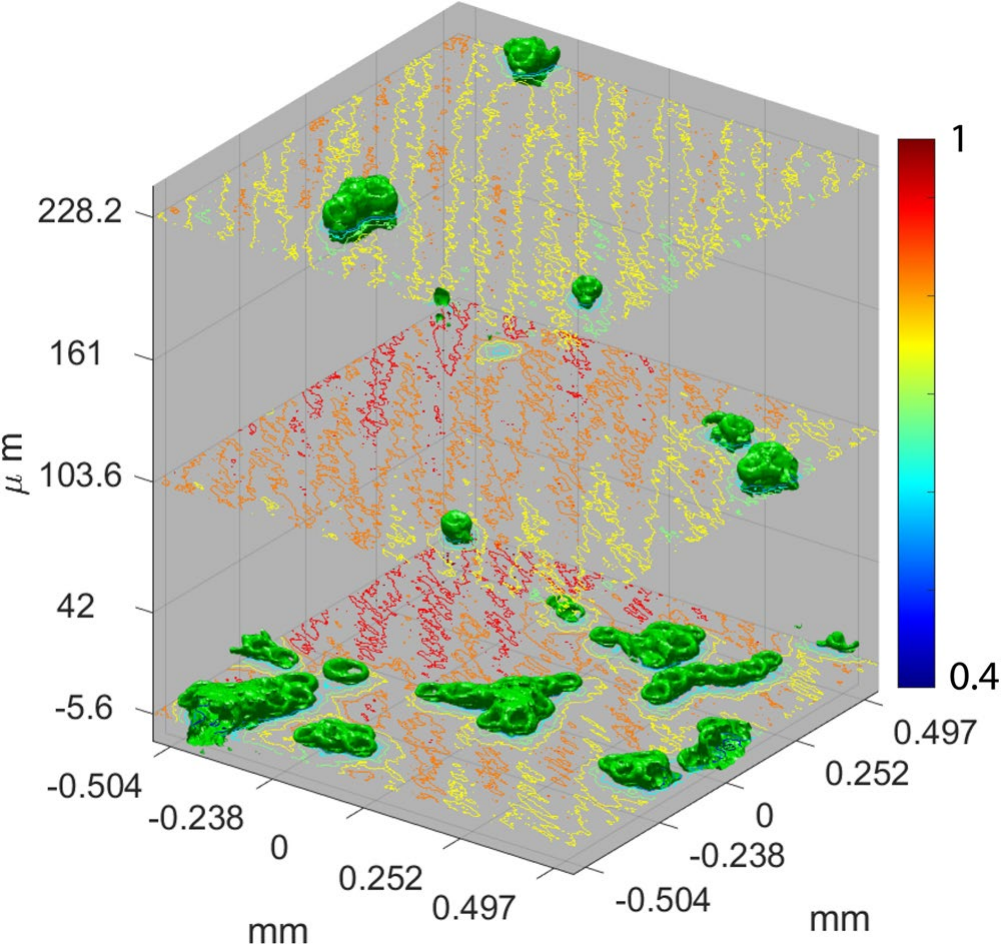
3-D view of latex spheres. Isosurface for experimental results of imaging of latex spheres along 222 longitudinal planes in a region corresponding to the red square in Fig. 4.

Figure 5 shows an isosurface (green) for the intensity of 0.54 from normalized intensity patterns. It was built from images measured along the longitudinal position and numbered from 1 to 222. Contours of intensity varying from 0.4 to 1 are shown for planes of number 16, 94, 183. The images correspond to the region marked by a red square in Fig. 4. The longitudinal planes are marked from the bottom to the top with the $z=0$ corresponding to the first image shown in Fig. 4, which is the plane of number 20.

## Discussion

Since the speckle field can be seen as the interference of plane waves with several random wave vectors, scattering by particles in a medium does not affect the random nature of the field. Even after scattering by an inhomogeneous medium, the average intensity continues to be homogenous and constant after the reconfiguration distance. For this reason, it is possible to envisage a new 3-D type of microscope, using speckles whose mean intensities are similar to self-reconstructing beams^2^. Notice that, the reconstructing distance depends fundamentally on the coherence length. Since the coherence length can be reduced beyond the diffraction limit of a focusing objective, we can strongly enhance not only the transversal resolution^18^, but also the axial resolution for imaging through scattering media. Our approach is insensitive to sample or aberration-induced illumination deformations. It does not require any reconstruction algorithm or numerical correlation as in ref.^25^. In addition, our findings are suitable for transmission of quantum and classical information through opaque obstacles.

## Electronic supplementary material

Supporting Information

Supporting Information
