# Supplementary material for: Three-Dimensional Speckle Light Self-Healing-Based Imaging System
Source: Sci Rep. 2018 Jan 12;8:563. doi: 10.1038/s41598-017-18952-0 (PMC5766565; doi:10.1038/s41598-017-18952-0)
Supplement: Supplementary file 1 — Supporting Information [file 41598_2017_18952_MOESM1_ESM.doc]

Supporting Information for “Three-Dimensional Speckle Light Self-Healing-Based Imaging System”

Danilo G. Pires^1^, Artur F. Sonsin^1^, Alcenísio J. Jesus-Silva^1^, and Eduardo J. S. Fonseca^1^

^1^Instituto de Física, Universidade Federal de Alagoas, Maceio, Alagoas 57061-970, Brazil

**I. Theory**

This theory was coined for the configuration in Fig. 2(a), but contain the basic operating principle of the idea in this paper. The field impinging on the RGGD is a field whose transversal profile is Gaussian,

$$E_{1}\left(\vec{r}_{1}\right)=\exp\left(-r_{1}^{2}/w_{0}^{2}\right)\exp\left(i\psi\right),$$

where$w_{0}$is the beam waist and $\psi$ its phase due the first lens. The description of the field propagation from the RGGD to the first obstacle (square) is given by a Fourier transform,

$E_{2}\left(\vec{r}_{2}\right)=\int E_{1}\left(\vec{r}_{1}\right)G\left(\vec{r}_{1}\right)\exp\left(\frac{i2\pi}{\lambda f_{2}}\vec{r}_{1}\cdot\vec{r}_{2}\right)d^{2}r_{1},$ (2)

where $G\left(\vec{r}_{1}\right)$ describes de effect of the RGGD and $\lambda$ is the wavelength. Successively applying the Colins’ integral formula^1^ we can describe the field propagation between the obstacles and lenses to the CCD camera. To make use of it, we need to describe the ABCD ray matrix of the system. The matrices for the propagation between the obstacles describes just a propagation through air by a distance $d$:

$\left[\begin{matrix} A & B \\ C & D \end{matrix}\right]=\left[\begin{matrix} 1 & d \\ 0 & 1 \end{matrix}\right]$ (3)

And the matrix for the propagation from the last obstacle to the CCD camera includes the propagation through the air and both lenses $L_{3}$and $L_{4}$until the light reaches the CCD. In this case, we have:

$\left[\begin{matrix} A & B \\ C & D \end{matrix}\right]=\left[\begin{matrix} 1 & f_{2}+z \\ 0 & 1 \end{matrix}\right]\left[\begin{matrix} 1 & 0 \\ -1/f_{2} & 1 \end{matrix}\right]\left[\begin{matrix} 1 & 2f_{2} \\ 0 & 1 \end{matrix}\right]\left[\begin{matrix} 1 & 0 \\ -1/f_{2} & 1 \end{matrix}\right]\left[\begin{matrix} 1 & f_{2}-2d \\ 0 & 1 \end{matrix}\right]=\left[\begin{matrix} -1 & 2d-z \\ 0 & -1 \end{matrix}\right]$ (4)

After obtain the elements of the ABCD ray matrices, we propagate the field through the planes $r_{i}$ in the experimental setup (See Fig. 2(a)). The Colins’ integral formula can be expressed as^1^:

$E_{i}(\overrightarrow{r_{i}})=\frac{ik}{2\pi B}\exp(-ikL)\int E_{j}(\overrightarrow{r_{j}})\exp\left\{\left(-\frac{ik}{2B}\right)\left[D\left|\overrightarrow{r_{i}}\right|^{2}-2\overrightarrow{r_{i}}\cdot\overrightarrow{r_{j}}+A\left|\overrightarrow{r_{j}}\right|^{2}\right]\right\}d^{2}r_{j},$ (5)

where *A,B,D* are the ABCD ray matrix elements for a propagation of length *L* from the transverse plane $r_{i}$ to $r_{j}$. The result for the field at the CCD camera is,

$\begin{matrix} E_{5}\left(\vec{r}_{5}\right)=\int S\left(\vec{r}_{4}\right)\exp\left[\frac{i\pi}{\lambda\left(2d-z\right)}\left(\vec{r}_{4}+\vec{r}_{5}\right)^{2}\right]\int T\left(\vec{r}_{3}\right)\exp\left[-\frac{i\pi}{\lambda d}\left(\vec{r}_{3}-\vec{r}_{4}\right)^{2}\right] \\ \times\int D\left(\vec{r}_{2}\right)\exp\left[-\frac{i\pi}{\lambda d}\left(\vec{r}_{2}-\vec{r}_{3}\right)^{2}\right]\int E_{1}\left(\vec{r}_{1}\right)G\left(\vec{r}_{1}\right)\exp\left(\frac{i2\pi}{\lambda f_{2}}\vec{r}_{1}\cdot\vec{r}_{2}\right)d^{2}r_{1}d^{2}r_{2}d^{2}r_{3}d^{2}r_{4}, \end{matrix}$ (6)

where $S\left(\vec{r}_{4}\right)$, $T\left(\vec{r}_{3}\right)$and $D\left(\vec{r}_{2}\right)$represent the square, triangular and diamond shaped obstacles, respectively, and $z$is the displacement of the CCD camera from its initial position at the focus of $L_{4}$. The mean intensity $I\left(\vec{r}_{5}\right)=\left\langle E_{5}\left(\vec{r}_{5}\right)E_{5}^{*}\left(\vec{r}_{5}\right)\right\rangle$ detected by the CCD camera is given by,

$\begin{matrix} I\left(\vec{r}_{5}\right)=\left\langle\int\int S\left(\vec{r}_{4}\right)S^{*}\left(\vec{r}'_{4}\right)\right.\exp\left\{\frac{i\pi}{\lambda\left(2d-z\right)}\left[\left(\vec{r}_{4}+\vec{r}_{5}\right)^{2}-\left(\vec{r}'_{4}+\vec{r}_{5}\right)^{2}\right]\right\} \\ \times\int\int T\left(\vec{r}_{3}\right)T^{*}\left(\vec{r}'_{3}\right)\exp\left\{-\frac{i\pi}{\lambda d}\left[\left(\vec{r}_{3}-\vec{r}_{4}\right)^{2}-\left(\vec{r}'_{3}-\vec{r}'_{4}\right)^{2}\right]\right\} \\ \times\int\int D\left(\vec{r}_{2}\right)D^{*}\left(\vec{r}'_{2}\right)\exp\left\{-\frac{i\pi}{\lambda d}\left[\left(\vec{r}_{2}-\vec{r}_{3}\right)^{2}-\left(\vec{r}'_{2}-\vec{r}'_{3}\right)^{2}\right]\right\}\int\int E\left(\vec{r}_{1}\right)E^{*}\left(\vec{r}'_{1}\right)G\left(\vec{r}_{1}\right)G^{*}\left(\vec{r}'_{1}\right) \\ \left.\times\exp\left[\frac{i2\pi}{\lambda f_{2}}\left(\vec{r}_{1}\cdot\vec{r}_{2}-\vec{r}'_{1}\cdot\vec{r}'_{2}\right)\right]d^{2}r_{1}d^{2}r_{2}d^{2}r_{3}d^{2}r_{4}d^{2}r'_{1}d^{2}r'_{2}d^{2}r'_{3}d^{2}r'_{4}\right\rangle. \end{matrix}$ (7)

The average in Eq. (7) is over the random effect $G\left(\vec{r}_{1}\right)$ of the RGGD, resulting $\left\langle G\left(\vec{r}_{1}\right)G^{*}\left(\vec{r}'_{1}\right)\right\rangle=\delta\left(\vec{r}_{1}-\vec{r}'_{1}\right)$^2,3^. The integral in $\vec{r}_{1}$is written as,

$\int\left|E\left(\vec{r}_{1}\right)\right|^{2}\exp\left[\frac{i2\pi}{\lambda f_{2}}\vec{r}_{1}\cdot\left(\vec{r}_{2}-\vec{r}'_{2}\right)\right]d^{2}r_{1}=\exp\left[-\frac{\left(\vec{r}_{2}-\vec{r}'_{2}\right)^{2}}{2\delta^{2}}\right],$ (8)

where $\delta=\lambda f_{2}/\left(\pi w_{0}\right)$ is the coherence length^4^. For small $\delta$ we can approximate the result in Eq. (8) by a Dirac’s delta function$\delta\left(\vec{r}_{2}-\vec{r}'_{2}\right)$. Inserting this result in Eq. (7) we can evaluate the integral in $\vec{r}'_{2}$ and Eq. (7) can be written in a simpler form,

$$\begin{matrix} I\left(\vec{r}_{5}\right)=\int\int S\left(\vec{r}_{4}\right)S^{*}\left(\vec{r}'_{4}\right)\exp\left[\frac{i\pi}{\lambda\left(2d-z\right)}\left(r_{4}^{2}+2\vec{r}_{4}\cdot\vec{r}_{5}-\left.r'_{4}^{2}-2\vec{r}'_{4}\cdot\vec{r}_{5}\right)\right.\right] \\ \int\int T\left(\vec{r}_{3}\right)T^{*}\left(\vec{r}'_{3}\right)\exp\left[-\frac{i\pi}{\lambda d}\left(r_{3}^{2}-2\vec{r}_{3}\cdot\vec{r}_{4}+r_{4}^{2}-r'_{3}^{2}+2\vec{r}'_{3}\cdot\vec{r}'_{4}-r'_{4}^{2}\right)\right] \\ \int D\left(\vec{r}_{2}\right)\exp\left\{-\frac{i\pi}{\lambda d}\left[2\vec{r}_{2}\cdot\left(\vec{r}'_{3}-\vec{r}_{3}+r_{3}^{2}-r'_{3}^{2}\right)\right]\right\}d^{2}r_{2}d^{2}r_{3}d^{2}r'_{3}d^{2}r_{4}d^{2}r'_{4}. \end{matrix}$$

(9)

The final result of Eq. (9), after the evaluation of all integrals, depends on the value of $z.$First consider the case $z=0$. Using the change of variables $\vec{r}'_{3}=\vec{r}+\vec{r}_{3}$, we notice that the integral in $\vec{r}_{3}$ can be written as,

$\begin{matrix} \int T\left(\vec{r}_{3}\right)T^{*}\left(\vec{r}+\vec{r}_{3}\right)\exp\left\{-\frac{i\pi}{\lambda d}\left[-2\vec{r}_{3}\cdot\left(\vec{r}_{4}-\vec{r}'_{4}+2\vec{r}\right)\right]\right\}d^{2}r_{3} \\ \approx\delta\left(\vec{r}_{4}-\vec{r}'_{4}+2\vec{r}\right), \end{matrix}$ (10)

being approximated by a Dirac’s delta function, since quadratic terms in $\vec{r}_{3}$ does not appear in the exponential and we are making a Fourier transform of an almost everywhere constant function^5^. The function value in the obstacle itself is 0, and in the much bigger outside region is 1. We claim that this argument is also valid for the others obstacles. Therefore, performing the integral in $\vec{r}'_{4}$ and the change of variables $\vec{r}'_{4}=\vec{r}_{4}+2\vec{r}$, the following result is obtained,

$I\left(\vec{r}_{5}\right)=\int\int\int D\left(\vec{r}_{2}\right)S\left(\vec{r}_{4}\right)S^{*}\left(\vec{r}_{4}+2\vec{r}\right)\exp\left[-\frac{i2\pi}{\lambda d}\vec{r}\cdot\left(\vec{r}_{2}+\vec{r}_{5}\right)\right]d^{2}r_{4}d^{2}rd^{2}r_{2}$ (11)

where the integral in $\vec{r}$ also can be approximated by a Dirac’s delta function,

$\int S^{*}\left(\vec{r}_{4}+2\vec{r}\right)\exp\left[-\frac{i2\pi}{\lambda d}\vec{r}\cdot\left(\vec{r}_{2}+\vec{r}_{5}\right)\right]d^{2}r\approx\delta\left(\vec{r}_{2}+\vec{r}_{5}\right).$ (12)

Finally, using Eq. (12) in Eq. (11), we arrive at the result,

$I\left(\vec{r}_{5}\right)=\int S\left(\vec{r}_{4}\right)d^{2}r_{4}\int D\left(\vec{r}_{2}\right)\delta\left(\vec{r}_{2}+\vec{r}_{5}\right)d^{2}r_{2}\propto D\left(-\vec{r}_{5}\right).$ (13)

Therefore, Eq. (13) shows that an inverted image of unit magnification of the diamond shaped obstacle is observed at $z=0$.

Let’s consider now the case $z=d$in Eq. (9). In this case, the integrals in $\vec{r}_{4}$ and $\vec{r}'_{4}$can immediately be approximated by a Dirac’s delta functions, since the quadratic terms in $\vec{r}_{4}$and $\vec{r}'_{4}$does not appear in the exponential,

$\begin{matrix} \int S\left(\vec{r}_{4}\right)\exp\left[\frac{i2\pi}{\lambda d}\vec{r}_{4}\cdot\left(\vec{r}_{3}+\vec{r}_{5}\right)\right]d^{2}r_{4}\approx\delta\left(\vec{r}_{3}+\vec{r}_{5}\right) \\ \int S^{*}\left(\vec{r}'_{4}\right)\exp\left[-\frac{i2\pi}{\lambda d}\vec{r}'_{4}\cdot\left(\vec{r}'_{3}+\vec{r}_{5}\right)\right]d^{2}r'_{4}\approx\delta\left(\vec{r}'_{3}+\vec{r}_{5}\right), \end{matrix}$ (14)

After performing these approximations the integrals in $\vec{r}_{3}$and $\vec{r}'_{3}$ can be evaluated, resulting

$I\left(\vec{r}_{5}\right)=T\left(-\vec{r}_{5}\right)T^{*}\left(-\vec{r}_{5}\right)\int D\left(\vec{r}_{2}\right)d^{2}r_{2}\propto T\left(-\vec{r}_{5}\right).$ (15)

Equation (15) shows that an inverted image of unit magnification of the triangular obstacle appears at$z=d$.

Finally, let’s consider the case $z=2d$. The first exponential appearing in Eq. (9) has a helpful limit in this case,

$\lim_{z\to2d} \exp\left\{\frac{i\pi}{\lambda\left(2d-z\right)}\left[\left(\vec{r}_{4}+\vec{r}_{5}\right)^{2}-\left(\vec{r}'_{4}+\vec{r}_{5}\right)^{2}\right]\right\}=\delta\left(\vec{r}_{4}+\vec{r}_{5},\vec{r}'_{4}+\vec{r}_{5}\right)$ (16)

Substituting Eq. (16) in Eq. (9), performing the integrals in the variables $\vec{r}_{4}$and $\vec{r}'_{4}$ and the change of variables $\vec{r}'_{3}=\vec{r}+\vec{r}_{3}$, results in

$\begin{matrix} I\left(\vec{r}_{5}\right)\propto S\left(-\vec{r}_{5}\right)\int\int T\left(\vec{r}_{3}\right)T^{*}\left(\vec{r}+\vec{r}_{3}\right)\exp\left(\frac{i4\pi}{\lambda d}\vec{r}\cdot\vec{r}_{3}\right)d^{2}r_{3} \\ \int D\left(\vec{r}_{2}\right)\exp\left[-\frac{i\pi}{\lambda d}\left(-2r^{2}+2\vec{r}\cdot\vec{r}_{5}+2\vec{r}\cdot\vec{r}_{2}\right)\right]d^{2}r_{2}d^{2}r, \end{matrix}$ (17)

where the integral in $\vec{r}_{3}$can be approximated by $\delta\left(\vec{r}\right)$. Therefore, the final result is,

$I\left(\vec{r}_{5}\right)\propto S\left(-\vec{r}_{5}\right).$ (18)

Eq. (18) shows that an inverted image of unit magnification of the square obstacle appears at$z=2d$.

**Supplementary References:**

1. Collins, S. A. Lens-System Diffraction Integral Written in Terms of Matrix Optics*. *J. Opt. Soc. Am.* **60**, 1168-1177 (1970).
2. Mandel, L. & Wolf, E. *Optical coherence and quantum optics*. (Cambridge University Press, 1995).
3. Alves, C. R., Jesus-Silva, A. J. & Fonseca, E. J. S. Effect of the spatial coherence length on the self-reconfiguration of a speckle field. *Phys. Rev. A* **94**, 013835 (2016).
4. Wang, F., Liu, X., Yuan, Y. & Cai, Y. Experimental generation of partially coherent beams with different complex degrees of coherence. *Opt. Lett.* **38**, 1814-1816 (2013).
5. Alves, C. R., Jesus-Silva, A. J. & Fonseca, E. J. S. Using speckles to recover an image after its transmission through obstacles. *Phys. Rev. A* **93**, 043816 (2016).
